# Microglial Engulfment of Spines in the Ventral Zona Incerta Regulates Anxiety-Like Behaviors in a Mouse Model of Acute Pain

**DOI:** 10.3389/fncel.2022.898346

**Published:** 2022-07-14

**Authors:** Zahra Farzinpour, An Liu, Peng Cao, Yu Mao, Zhi Zhang, Yan Jin

**Affiliations:** ^1^Department of Anesthesiology and Pain Medicine, The First Affiliated Hospital of USTC, Division of Life Sciences and Medicine, University of Science and Technology of China, Hefei, China; ^2^Department of Physiology, School of Basic Medical Sciences, Anhui Medical University, Hefei, China; ^3^Stroke Center and Department of Neurology, The First Affiliated Hospital of USTC, Division of Life Sciences and Medicine, University of Science and Technology of China, Hefei, China

**Keywords:** anxiety-like behaviors in pain, inflammatory pain model, microglial engulfment, dendritic spines, GABAergic neurons, zona incerta, chemogenetic manipulation

## Abstract

Although activation of microglial cells is critical in developing brain disorders, their role in anxiety-like behaviors in pain is still vague. This study indicates that alteration of microglia’s neuronal spine engulfment capacity in ventral zona incerta (ZI_*V*_) leads to significant pain and anxiety-like behaviors in mice 1-day post-injection of Complete Freud’s Adjuvant (CFA1D). Performing whole-cell patch-clamp recordings in GABAergic neurons in the ZI_*V*_ (ZI_*V*_*^GABA^*) in brain slices, we observed decreased activity in ZIv*^GABA^* and reduced frequency of the miniature excitatory postsynaptic currents (mEPSCs) in ZI_*V*_*^GABA^* of CFA1D mice compared with the saline1D mice. Besides, chemogenetic activation of ZI_*V*_*^GABA^* significantly relieved pain and anxiety-like behaviors in CFA1D mice. Conversely, in naïve mice, chemogenetic inhibition of ZI_*V*_*^GABA^* induced pain and anxiety-like behaviors. Interestingly, we found changes in the density and morphology of ZI_*V*_*^Microglia^* and increased microglial engulfment of spines in ZI_*V*_ of CFA1D mice. Furthermore, pain sensitization and anxiety-like behaviors were reversed when the ZI_*V*_*^Microglia^* of CFA1D-treated mice were chemically inhibited by intra-ZI_*V*_ minocycline injection, accompanied by the recovery of decreased ZI_*V*_*^GABA^* excitability. Conclusively, our results provide novel insights that dysregulation of microglial engulfment capacity encodes maladaptation of ZI_*V*_*^GABA^*, thus promoting the development of anxiety-like behaviors in acute pain.

## Introduction

Besides its impact on physical function, pain sensitivity notably affects mental well-being and quality of life. Moreover, one of the most common psychological disorders in adulthood is the anxiety (Bandelow and Michaelis, 2015), strongly associated with pain hypersensitivity. Clinically, patients suffering from pain have an intensified possibility of emerging anxiety, creating comorbid anxiety symptoms in the pain (Wang et al., 2015). Understanding the mechanism underlying this cycle of pain and anxiety symptoms is crucial for developing treatment options for anxiety-like behaviors in pain.

Brain areas involving sensory pathways such as the spinothalamic tract send extreme nociceptive outputs to the zona incerta (ZI) (Wang et al., 2020), which is related to many behaviors, including the pain (Masri et al., 2009; Petronilho et al., 2012; Moon et al., 2016; Moon and Park, 2017). Several studies demonstrate that the ZI is a critical locus in pain processing; the antinociceptive effect of ZI is probably caused by the activation of a pain-inhibitory mechanism (Petronilho et al., 2012) since decreased neuronal activity in the ZI generates neuropathic pain (Moon et al., 2016; Moon and Park, 2017). These studies have shown that neuropathic pain is associated with a reduction in neuronal activity of the GABAergic neurons in the ZI (ZI*^GABA^*) in rat nerve chronic constriction injury (CCI) (Moon and Park, 2017) and spinal cord injury (SCI) models (Moon et al., 2016). Repeated motor cortex stimulation (MCS) intensively implicates the ZI as a nucleus of maladaptive plasticity in the chronic pain modulation (Hu et al., 2019), primarily mediated by projections to ZI*^GABA^*. In an animal model of neuropathic pain, 50–60 Hz deep brain stimulation of ZI reduces the hyperalgesia (Lucas et al., 2011). Remarkably, in human subjects, stimulation of ZI achieves a moderate but significant analgesic effect (Lu et al., 2021).

In addition to the role of ZI and its neuronal subpopulations in the human anxiety (Burrows et al., 2012), recently, optogenetic manipulations of the ZI neurons in an animal model triggered anxiety-like behaviors (Li et al., 2021). Activation of ZI or targeted stimulation of ZI*^GABA^* decreases fear generalization and enhances the extinction recall (Zhou H. et al., 2021). Moreover, parvalbumin-expressing neurons in ZI have an essential role in promoting the nocifensive behaviors (Wang et al., 2020) and itch modulation (Li et al., 2022). Therefore, it would be valuable to decipher the ZI_*V*_*^GABA^* alterations in the mouse model of anxiety-like behavior in acute pain.

Though microglia display an active and dynamic phenotype during neuroinflammation, whether and how microglia regulate anxiety-like behaviors in pain remains mysterious. Microglia cells are responsible for physiological functions such as neuronal regulation, synapse pruning, and clearance of cellular and toxic debris (Kisler et al., 2021). Microglial cells quickly respond to any slight modification in the central nervous system (CNS) (Nimmerjahn et al., 2005; Raivich, 2005). Chronic conditions such as stress affect microglia density, morphology, and molecular signature, indicating an altered function (Picard et al., 2021). These microglial alterations lead to synaptic plasticity and cognitive and behavioral consequences. Microglial inhibitors such as minocycline provide a protective effect and modulate microglia reactivity and polarization (Ahmed et al., 2017). There is accumulating evidence that reveals a relationship between anxiety and pain behavior and microglia activity, showing that microglial inhibitors can lessen microgliosis, alleviate the development of pain sensitivity and ameliorate different comorbidity of pain (Rojewska et al., 2014) and anxiety (Rooney et al., 2020).

In this study, using the mouse model of anxiety-like behavior in pain induced by complete Freund’s adjuvant (CFA), combining chemogenetic, electrophysiological recording, pharmacological techniques, 3D reconstruction of microglial cells, and qPCR analysis, we examined the considered significance of microglia through actions on ZIv*^GABA^* adaptation in a manner that later promotes anxiety-like behaviors in pain and anxiety. Taken together, we described the importance of microglia as a critical regulator in anxiety-like behavior in pain through actions on ZI_*V*_*^GABA^* adaptation.

## Materials and Methods

### Mouse Models

Animals had acclimatized 3 days before the beginning of the experimental procedures. They were treated under the requirements of the Animal Care and Use Committee of the University of Science and Technology of China. In these experiments, *C57BL/6J*, *GAD2-Cre*, and *Ai14 (RCL-tdT)* mice; were used at 8–10 weeks of age, supplied by GemPharmatech and Charles River or Jackson. The animals were housed under a 12 h light/dark cycle (lights on from 7:00 a.m. to 7:00 p.m.) at a constant temperature (23–25°C) with a relative humidity of 60 ± 5%. The animals’ behavior was recorded using a video tracking system and analyzed offline during the testing session. All efforts were made to minimize pain or discomfort, and the minimum number of animals was used.

Previous studies established the mouse model of anxiety-like behaviors in pain by hind paw CFA injection (Wang et al., 2015). In the present study, all mice were anesthetized with isoflurane (around 10 s) before injection of CFA (10 μL, Sigma-Aldrich, St. Louis, MO, United States) into the left hind paws, as explained (Zhou W. et al., 2021) previously. The control group received an identical injection of an equal amount of saline into the left hind paw.

### Histology, Immunohistochemistry, and Imaging and Image Analysis

After the trial, the animals have anesthetized with isoflurane (around 15 s) and consecutively perfused transcranially with 4% paraformaldehyde (PFA) (w/v) (4 min) and saline (3 min). Brains were successively apart and post-fixed in 4% PFA solution at 4°C overnight and next immersed in sucrose solution (w/v) (20% and 30%) at 4°C for 2−3 days to dehydrate. Frozen brains were cut into coronal sections (40 μm) with a cryostat microtome system (CM1860, Leica, Wetzlar, Germany) at –20°C, and slices were used for immunofluorescence. Animals with improper localization of needles or optical fibers’ tips were excluded from the study.

For staining, the ZI_*V*_ brain slices were washed three times with Phosphate-buffered saline (PBS) (pH 7.4) for 5 min and blocked with 10% donkey serum at room temperature for 1 h in PBS with 0.5% (v/v) Triton X-100. Sections were then incubated with primary antibodies, including rabbit anti-Iba-1 (1:500, 019-19741, Wako, Richmond, VA, United states; RRID: AB_839504), goat anti-PSD95 (1: 200, ab12093, AbCam, Cambridge, United Kingdom; RRID: AB_298846), mouse anti-MHCII (1: 500, ab23990, AbCam, Cambridge, United Kingdom; RRID: AB_447796), and mouse anti-CD68 (1: 500, ab955, AbCam, Cambridge, United Kingdom; RRID: AB_307338) at 4°C for 24 h with 3% Triton X-100 and donkey serum. Next, sections were washed three times with PBS for 5 min and incubated with secondary antibodies for 1.5 h at room temperature in a dark place. The following secondary antibodies were used: donkey anti-rabbit 488 (1: 500, A21206, Invitrogen, Waltham, MO, United States), donkey anti-goat 594 (1: 500, A11058, Invitrogen, Waltham, MO, United States), donkey anti-mouse 647 (1: 500, A31571, Invitrogen, Waltham, MO, United States). Finally, the sections were washed thrice, counterstained with DAPI (D9542, Sigma-Aldrich, St. Louis, MO, United States) dilution buffer (1: 2000) for 5 min, and washed twice in PBS. All the sections were mounted with an antifade mounting medium (Cat. No. H-1000, Vector Laboratories, Newark, CA, United States).

For quantification of immunohistochemistry analyses, slide images were acquired and visualized with microscopes (ZEISS, Jena, Germany LSM880 and Leica, Wetzlar, Germany DM2500). Two slices containing the ZI_*V*_ region picked from per mouse were imaged and quantified for five mice in each group for imaging. Analysis of cell counts using ImageJ software (Fiji edition, NIH) by an observer blind to the experimental conditions. Intensity colocalization was adjusted to count moderate- to highly immunoreactive cells accurately. A mean count per mouse was used for statistical evaluation.

### Behavioral Experiments

Behavioral experiments were performed to measure anxiety-like behaviors in pain in CFA treated- mice.

#### Von Frey Filament Test

A set of Von Frey monofilaments (Stoelting Inc., Chicago, IL, United States), the gold standard for determining mechanical thresholds, assesses mechanical nociception. After 40 min of habituation for three consecutive days, mice were placed in a Plexiglas cage (20 × 20 × 14 cm) within a grid bottom to assess the pain threshold. Stimulus intensity (g) is the strength of the sensory input to the brain and was evaluated by delivering a continuous pre-determined mechanical stimulus (with marking forces of 0.04, 0.16, 0.4, 0.6 g) for 2–5 s on the plantar region of the paws ipsilateral and contralateral to the CFA lesion. To record the stimulus intensity in response to the stimulus, each hind paw was measured five times at a 5- min interval, and the mean value was recorded.

#### Open Field Test

The open-field test (OFT) measures various behaviors in animals, for example, “spontaneous exploratory locomotion” and “the tendency of the animals to prefer the periphery rather than the center of the OFT” in an unknown chamber with four surrounding walls. The apparatus consisted of a large area composed of plastic, surrounded by 100 cm high walls. Each animal was gently placed in the center of the OFT, and its behavior was videotaped. The time spent in the center (mice, 50 × 50 × 25 cm) and the distance the animals traveled were measured for 6 min using EthoVision XT 14 software (Noldus, Wageningen, The Netherlands). After each test, the area was cleaned with 75% ethanol to remove olfactory cues from the apparatus. Heat maps also were created in EthoVision XT, where color codes show time spent in each spot.

#### Elevated Plus-Maze

The elevated plus-maze (EPM) consisted of four equally illuminated plastic arms (6 × 60 cm) radiating at square angles from a central platform 30 cm above the floor. Two opposite arms were enclosed by 30 cm high plastic walls, and the other two arms were open. For all behavioral assessments, animals were acclimatized at least 3 days before testing. The animals were released from the central platform, with their face pointing toward an enclosed arm. Animal behavior was recorded with a camera. The time spent in the open arms and the number of entries into the open arms were analyzed using EthoVision XT. The area was cleaned between tests using 75% ethanol. Heat maps also were created in EthoVision XT, where color codes show time spent in each spot.

### Electrophysiological Recordings

#### Brain Slices Preparation

The mice were anesthetized with isoflurane (around 15 s) followed by pentobarbital sodium (20 mg/kg, i.p.) and afterward intra-cardinally perfused with ∼20 mL oxygenated ice-cold *N*-Methyl-D-Glucamine (NMDG) and ACSF containing: 93 NMDG, 2.5 KCl, 1.2 NaH_2_PO_4_, 30 NaHCO_3_, 20 *N*-2-hydroxyethyl piperazine-*N*-2-ethane sulfonic acid (HEPES), 25 glucoses, 2 thiourea, 5 Na-ascorbate, 3 Na-pyruvate, 0.5 CaCl_2_, 10 MgSO_4_, and 3 glutathione (GSH) (in Mm, PH: 7.3–7.4, the osmolality: 300–305 mOsm). After perfusion, the brain slices (300 μm) containing ZI_*V*_ were quickly sectioned by chilled (2−4°C) NMDG ACSF on a microtome (VT1200s, Leica, Germany) vibrating at 0.18 mm/s velocity. The brain slices were quickly incubated in oxygenated NMDG ACSF (saturated with 95% O_2_/5% CO_2_ to provide stable pH and continuous oxygenation) for 10–12 min at 33°C and then for at least 1 h at 25°C incubated in HEPES ACSF containing; 92 NaCl, 2.5 KCl, 1.2 NaH_2_PO_4_, 30 NaHCO_3_, 20 HEPES, 25 glucose, 2 thiourea, 5 Na-ascorbate, 3 Na-pyruvate, 2 CaCl_2_, 2 MgSO_4_, and 3 GSH (in Mm, PH: 7.3–7.4, the osmolality: 300–310 mOsm). Then the brain slices were transferred to a slice chamber (Warner Instruments, Holliston, MO, United States) for electrophysiological recording with continuous perfusion with standard ACSF that contained 129 NaCl, 3 KCl, 2.4 CaCl_2_, 20 NaHCO_3_, 10 glucose, 1.3 MgSO_4_, and 1.2 KH_2_PO_4_ (in mM, pH: 7.3−7.4, osmolality: 300−310 mOsm). The slices were moved to the chamber that was constantly perfused with oxygenated standard ACSF containing 124 NaCl, 2.4 CaCl_2_, 5 KCl, 1.3 MgSO_4_, 26.2 NaHCO_3_, 1.2 KH_2_PO_4_, and 10 glucose (in mM, pH: 7.3–7.4 osmolarity: 300–305 mOsm/kg) for recording at 33°C by solution heater (TC-344B, Warner Instruments, Holliston, MO, United States). The observer was blinded to the experimental purposes during recording and analyses.

#### Whole-Cell Patch-Clamp Recordings

Neurons were visualized using an infrared (IR)-differential interference contrast (DIC) microscope (BX51WI, Olympus, Tokyo, Japan) with a 40 × water-immersion objective. Whole-cell patch-clamp recordings were performed using patch pipettes (5−8 MΩ) that were pulled from borosilicate glass capillaries (VitalSense Scientific Instruments Co., Ltd., Wuhan, China) with an outer diameter of 1.5 mm on a four-stage horizontal puller (P-1000, Sutter Instruments, Novato, CA, United States). The signals were collected using a MultiClamp 700B amplifier, low-pass filtered at 2.8 kHz, digitized at 10 kHz with a Digidata 1440 A Data Acquisition System, and analyzed using pClamp10.7 (Molecular Devices, Sunnyvale, CA, United States). Neurons were excluded with a series resistance of more than 30 MΩ or that changed by more than 20% during the recording. To record the intrinsic membrane properties, micropipettes are filled with an internal solution containing K^+^. The intracellular solution contained (in mM): 5 KCl, 2 MgCl_2_, 10 HEPES, 2 Mg-ATP, 130 K-gluconate, 0.3 Na-GTP and 0.6 EGTA (pH: 7.4, osmolarity: 290−300 mOsm). For the recording of action potentials, in current-clamp mode, by injecting a known current amplitude to the inside of the cell through their setup and observing the change in cellular excitability in response to these current injections. There is a positive relationship between current injection, and the firing rate of the cells, alteration in intrinsic excitability is assessed through changes in the capability for neurons to generate action potentials. The rheobase was defined as the minimum current to elicit an action potential. Miniature excitability postsynaptic current (mEPSC) was recorded in the presence of tetrodotoxin (TTX, 1 mM, obtained from Hebei Aquatic Science and Technology Development Company, Hebei, China) and picrotoxin (PTX, 50 mM) at –70 mV. Unless otherwise stated, all drugs used for electrophysiological recording were purchased from Sigma- Aldrich, St. Louis, MO, United States.

### Stereotaxic Surgeries and Microinjections

#### Implantation of the Guide Cannula

The mice were anesthetized at 2% (w/v) pentobarbital sodium (50 mg/kg i.p.) and then placed in a stereotaxic frame (RWD Life Science Inc). A heating pad was used to preserve the temperature of the animal body at 36°C, and sterilized ointment was applied to the eyes to prevent corneal dehydration. A stainless steel guide cannula (outer diameter, 0.5 mm O.D. 0.48 mm-26G/M3.5, Shenzhen RWD Life Science Co., Ltd., China) was implanted unilaterally in the ZI_*V*_ at the following coordinates: –2.2 mm anterior to bregma, 1.50 mm lateral to the midline, and 4.2 mm below the surface of the brain according to the atlas of Paxinos and Watson (Paxinos and Franklin, 2019). After implantation of the guide cannula, a dummy cannula (outer diameter, 0.5 mm, Shenzhen RWD Life Science Co., Ltd., China) was carefully inserted and checked daily to prevent possible clogging. At least two skull screws were used for securing the guide cannula to the skull surface with dental acrylic.

#### Local Drug Infusion

The mice were anesthetized with isoflurane (around 15 s). An internal stainless-steel injector attached to a 10 μL syringe and a microsyringe pump (UMP3, WPI, Houston, TX, United States) was inserted into the guide cannula and used to infuse minocycline artificial cerebrospinal fluid (ACSF) as a vehicle into the ZI_*V*_. Drugs were dissolved in ACSF and injected in a volume of 0.3 μL at a 30 nL/min rate. The injection needle, 0.5 mm lower than the guide cannula, was used for microinjection with a polyethylene catheter connecting a micro-syringe. The mice were allowed at least 7 days for recovery before injections to minimize stress during the behavioral assays, and the mice with missed injections were excluded from the study.

#### Minocycline Preparation

Minocycline hydrochloride (Cat. No. M9511, Sigma-Aldrich, St Louis, MO, United States) is a second-generation semisynthetic tetracycline and could inhibit microglial cells. To study whether minocycline can prevent the development of anxiety-like behavior in pain following CFA injection, it was ready in ACSF as a 10 μg/μL solution. It was locally delivered by intra- ZI_*V*_ injection (0.3 μL in the contralateral hemisphere) at a 0.1 μL/min rate. A control group of mice received injections of equal amounts of ACSF. For electrophysiological recording, minocycline was prepared as a 10 mg/mL solution in 0.9% saline and administered (50 mg/kg, i.p.). Mice were pretreated with saline or minocycline 2 h before CFA injection and continued one more injection 30 min before electrophysiological recording. A control group of mice received injections of equal amounts of saline similarly.

### Chemogenetic Behavioral Testing of *GAD2-Cre* Mice

#### Surgery and Adeno-Associated Virus Injection

Naive Male, 8- to 12-week-old *GAD2-Cre* mice were anesthetized with pentobarbital (20 mg/kg) and then positioned in a stereotaxic frame (RWD Life Science Inc). A heating pad was used to maintain the core body temperature of the animals at 36°C. The skin’s surface was sanitized with 70% (w/vol) ethanol, the skull was cleaned, and a volume of 0.3 μL virus into the ZI_*V*_ (–2.2 mm anterior to bregma, 1.5 mm lateral to the midline, and 4.6 mm below the surface of the brain) using a glass micropipette (tip diameter 10–20 μm) attached to a 10 μL microsyringe pump (UMP3, WPI, Houston, TX, United States) at a rate of 30 nL/min. The rAAV-Ef1α-DIO-hM4D(Gi)-mCherry-WPRE-pA (AAV-hM4Di-mCherry), rAAV-Ef1α-DIO-hM3D (Gq)-mCherry-WPRE-pA (AAV-hM3Dq-mCherry) and rAAV-Ef1α- DIO-mCherry-WPRE-hGH-pA (AAV-mCherry) were purchased from BrainVTA, Wuhan, China. The Adeno-Associated Virus (AAV)-sparse-CSSP-YFP-8E3 virus is produced by co-packaging the AAV-CMV-DIO-Flp plasmid, and AAV-FDIO-EYFP plasmid with the ratio of 1:8E3 in a single rAAV production step (5.3 × 10^13^ vg/mL) was purchased from BrainCase Co., Ltd., Shenzhen, China.

According to Paxinos and Watson’s atlas, the virus was delivered into ZI_*V*_ bilaterally. Following injection, the needle was left in place for five extra minutes and then slowly withdrawn. Finally, the incision was closed with sutures. Electrophysiological recordings and behavior experiments have been performed at least 21 days post-viral injection. Animals with missed injections were excluded. We investigated the role of ZI_*V*_*^GABA^* in the anxiety-like behavior in the acute pain model; we have used designer receptors exclusively activated by designer drugs (DREADs) by custom drugs; we divided *GAD2-Cre* mice into the activation group (AAV-DIO-hM3Dq-mCherry), inhibition group (AAV-DIO-hM4Di-mCherry), and control group (mCherry).

#### Clozapine *N*-Oxide for Designer Receptors Exclusively Activated by Designer Drugs Experiments

Clozapine *N*-oxide (CNO) (5 mg, Sigma- Aldrich, St. Louis, MO, United States) for designer receptors exclusively activated by designer drugs (DREADD) tests (MedChemExpress, Monmouth Junction, NJ, United States) was i.p. injected 30 min beforehand behavior assessments. Three weeks after viral injection, animals were injected with CNO (1 mg/kg, 0.5 mL, i.p.) an hour before starting behavioral examinations or electrophysiological recordings. Two weeks after the viral injection and 1 week before the CNO administration, the anxiety behaviors and mechanical sensitivity baselines were recorded. One-day post- CFA injection (CFA1D), the mechanical pain threshold and anxiety-like behaviors were tested, and the effect of CNO was observed and compared to mCherry. For chemogenetic experiments, adapt the mice 2 days before the experiments. On the day of the investigation, adapt the mice for 1 h, and inject CNO within 30 min. The mechanical pain threshold test is started after 30 min, and the test is performed every 10 min. After five tests, the experiment ends. The animal with missed injections was excluded from the study.

### Three-Dimensional (3D) Reconstruction of Iba-1^+^ Cells

Microglia were imaged from ZI_*V*_ but clearly outside the microinjection tract after minocycline or ACSF microinjection. Imaging was performed using a 40 × objective (1.3 numerical aperture), and imaging parameters (laser power, gain and offset) were consistent. Microscopic fields were randomly acquired as 512 × 512-pixel images (Z-step, 1.0 mm) in the mouse ZI_*V*_, and images were analyzed using Imaris 9.6.2 software (Bitplane, Zürich, Switzerland). The function of “Filaments” was used to quantify process length and number of branch points. Microglial engulfment was analyzed to create a 3D surface rendering of the microglia, with a threshold established to ensure accurate reconstruction of microglial processes, which was then used for subsequent reconstructions. The function of “Spots” was used to reconstruct the PSD95^+^ puncta. Finally, the Imaris MATLAB-based (MathWorks, Natick, MA, United States) plugin “Split into Surface Objects” was used to assess the number of PSD95^+^ puncta entirely within the microglial surface. For four mice, two images per mouse were reconstructed randomly in each group, with at least ten cells each. The mean result was used for morphological analysis.

Imaging was performed on a Zeiss LSM880 microscope using a 100 × 1.4 NA oil objective for microglial processes and neuronal dendrites contact analyses. The function of “Surface” was used to accurately reconstruct both Iba-1^+^ microglia and YFP^+^ dendrites, and initially established thresholds were then used for following reconstructions. Finally, the MATLAB-based Imaris plugin “Surface-Surface Contact Area” was used to measure the size of contact areas between microglial processes and neuronal dendrites.

### Quantitative PCR

The ZI_*V*_ tissue samples were dissociated from three CFA1D and saline1D mice. Total RNA was isolated and purified using the RNeasy kit (Qiagen), followed by reverse transcription using a Go-ScriptTM Reverse Transcription kit (Promega, A5001). Quantitative real-time PCR (qPCR) from 1 to 10 ng of cDNA templates was performed using an SYBR Green kit (GeneStar, Baltimore, MD, United States) on an ABI Step one system (Applied Biosystems, Waltham, MO, United States). *Gapdh* mRNA quantification was used as a loading control for normalization. Fold changes of mRNA levels over controls were calculated by the 2^–ΔΔ*CT*^ method using *Gapdh* as control. Each real-time PCR reaction was run in triplicates on a 96-well plate, and water was used as a negative control. Sequences of the primers (Sangon Biotech, Shanghai, China) used in PCR are listed as follows: TNFα (forward 5′-CCTGTAGCCCACGTCGTAG-3′, reverse 5′-GGGAGTAGACAAGGTACAACCC-3′); IL-1β (forward 5′-TTCAGGCAGGCAGTATCACTC-3′, reverse 5′-GAAGGTCCACGGGAAAGACAC-3′); or IL6 (forward 5′-GCCAACATTTTATTTCCGGGA-3′, reverse 5′-CCACTGAGCATATTTCTCGGG-3′).

### Statistical Analysis

The statistical analyses and graphing were performed by GraphPad Prism 8.0. (Graph Pad Software, Inc., San Diego, CA, United States). Statistical significance was defined as *P* < 0.05. We accompanied simple statistical comparisons using the Student’s *t*-test. ANOVA (one-way and two-way) and *post hoc* analyses were used to statistically analyze the experimental groups’ data with multiple comparisons. Unpaired or paired Student’s *t*-tests were used to compare the two groups. Significance levels are indicated as *P < 0.05, **P < 0.01, ***P < 0.001. ns represents not significant. All Behavioral experiments except for measuring anxiety-like behaviors (i.e., EPM and OFT) were performed at least twice. All data are expressed as the mean ± SEM.

## Results

### CFA1D Mice Demonstrate Anxiety-Like Behaviors in Acute Pain

**FIGURE 1:**
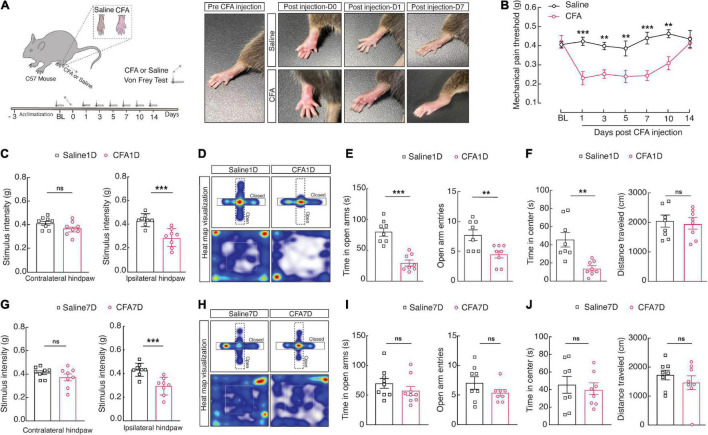
CFA1D mice demonstrate anxiety-like behaviors in acute pain. **(A)** Schematic diagram of the experiment paradigms for the CFA injection. **(B)** Time course of CFA-induced mechanical sensitivity on days 1, 3, 7, 10, and 14 post injection. Mechanical pain threshold; two-way ANOVA with Bonferroni *post hoc* analysis [Time × group interaction, *F*(5,854) = 3.930 and *P* = 0.0016]. **(C)** Performance of stimulus intensity of the contralateral and ipsilateral hind paw in saline1D- and -CFA1D mice (*t*_14_ = 1.741 and *P* = 0.1037 in contralateral hind paw, *t*_14_ = 4.393 and *P* = 0.006 in ipsilateral hind paw). Two-tailed unpaired *t*-test. **(D)** Representative animal heat tracks from saline1D- and CFA1D mice in EPM and OFT. **(E,F)** Performance of EPM and OFT on saline1D- and CFA1D mice (time in open arm; *t*_14_ = 6.200 and *P* < 0.001, open arm entries; *t*_14_ = 3.005 and *P* = 0.095, time in center; *t*_14_ = 3.76 and *P* = 0.0021, distance traveled; *t*_14_ = 0.56 and *P* = 0.58). **(G)** Performance of stimulus intensity of the contralateral and ipsilateral hind paw in saline7D- and CFA7D- mice (*t*_14_ = 1.185 and *P* = 0.2556 in contralateral hind paw, *t*_14_ = 4.193 and *P* = 0.0009 in ipsilateral hind paw). Two-tailed unpaired *t*-test. **(H)** Representative animal heat tracks from saline7D- and CFA7D mice in EPM and OFT. **(I,J)** Performance of EPM and OFT on saline7D- and CFA7D- mice (time in open arm; *t*_16_ = 1.118 and *P* = 0.2799, open arm entries; *t*_14_ = 1.414 and *P* = 0.1792, time in center; *t*_14_ = 0.5309 and *P* = 0.6038, distance traveled; *t*_14_ = 0.9066 and *P* = 0.38). All data are expressed as mean ± SEM. *n* = 8 mice per group. All data are expressed as mean ± SEM. **P < 0.01, and ***P < 0.001; ns, not significant.

Injection of complete Freund’s adjuvant (CFA) immediately induces pain sensitivity (Altarifi et al., 2019), and recovery occurs from 10 (Corder et al., 2013) to 14 days (Chang et al., 2010). Therefore, we design experiments to provide a time course for a single 10 μL injection of CFA-induced sensory pain on days 1, 3, 7, 10, and 14 post injections into the plantar hind paw (Figure 1A). Notably, at 1, 3, 7, and 10 days after CFA injection, the mice compared to the saline group displayed significant mechanical sensitivity in the von Frey test in the left paw (Figure 1B). These results suggest that the CFA injection is a reliable model for inducing mechanical sensitivity by the current inflammatory pain model.

Next, we established an animal model of anxiety-like behavior in pain through CFA injection in the left hind paw. One-day post-CFA (CFA1D) injection, the mice displayed significant pain and anxiety-like behaviors in the regular tests, including the EPM (i.e., decreased open arm entries and time in open arms) and the open field test (OFT) (i.e., reduced time in the center). The mechanical threshold for withdrawal caused by mechanical stimulus was significantly declined in CFA1D compared with saline1D mice in the ipsilateral hind paw, reflecting pain behaviors (Figure 1C). Interestingly, we found decreased open arm entries and time in open arms in the EPM and time in the center of the OFT (Figures 1D–F). Moreover, locomotor activity was not affected by CFA1D in this study (Figure 1F). It can be because this degree of pain did not interfere with the choice to continue running average distances (Cobos et al., 2012). Interestingly, withal significant pain hypersensitivity in 7-day post-CFA (CFA7D) mice (Figure 1G), we did not observe anxiety-like behavior nor during subsequent EPM and OFT measurements (Figures 1H–J). These findings propose that CFA1D mice provide a well-defined model to study anxiety-like behaviors in acute pain.

### CFA1D Mice Demonstrate a Decrease in the ZI_*V*_*^GABA^* Activity

An increase in the GABA release or activation of the GABA system may lessen several types of pathological pain (Wilke et al., 2020) or contrarily develop pain behaviors (Bravo-Hernández et al., 2014; Jang et al., 2017). A growing amount of evidence suggests that GABAergic interneurons could alleviate pain-related emotions; for example, muscimol microinjection into the ACC (Ang et al., 2015) and amygdala (Pedersen et al., 2007) could relieve the anxiety-like behaviors of CFA rats. So, we tested whether and how the GABAergic system in the ZI is involved in the CFA-induced pain model.

Zona incerta is engaged in various neuroplasticity types, including chronic pain regulation, and its activation inhibits the pain (Trageser et al., 2006; Masri et al., 2009; Petronilho et al., 2012; Moon et al., 2016; Moon and Park, 2017). As the ZI region mainly consists of GABAergic neurons (Masri et al., 2009), we performed whole-cell patch-clamp recordings in ZI_*V*_*^GABA^* in acute brain slices of the contralateral hemisphere to determine the neuronal activity in the ZI_*V*_ during anxiety-like behaviors in pain progression.

**FIGURE 2:**
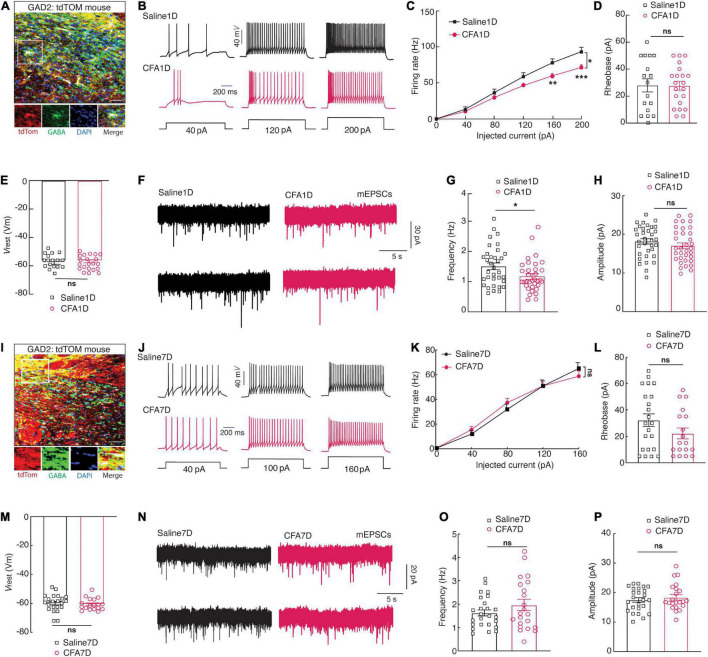
CFA1D mice demonstrate a decrease in the ZI_*V*_*^GABA^* activity. **(A)** Confocal images of ZI_*V*_*^GABA^* from *GAD-tdTOM* mice. The white box depicts the area shown in the box of the ZI_*V*_. Scale bar, 50 μm. **(B)** Sample traces and **(C)** summarized statistical data for action potential firing recorded from ZI_*V*_*^GABA^* in mice treated with saline1D or CFA1D. Two-way ANOVA with Bonferroni post-tests [Time × group interaction, *F*(5,180) = 6.735 and *P* < 0.05]. **(D–E)** The alterations in action potential properties in **(D)**, rheobase; *t*_36_ = 0.53 and *P* = 0.6012; **(E)** *V*_*rest*_ (mV); *t*_36_ = 0.527 and *P* = 0.6. **(F)** Sample traces of the mEPSCs from saline1D or CFA1D groups. **(G,H)** Summarized statistical data of **(G)** the frequency; *t*_66_ = 2.23 and *P* = 0.02; **(H)** the amplitude; *t*_66_ = 1.12 and *P* = 0.26 of CFA1D compared with saline1D mice. Action potential parameters were estimated from the initial evoked spike. *n* = 17–36 neurons per group. **(I)** Confocal images of ZI_*V*_*^GABA^* from *GAD-tdTOM* mice. The white box depicts the area shown in the box of the ZI_*V*_. Scale bar, 50 μm. **(J)** Sample traces and **(K)** summarized statistical data for action potential firing recorded from ZI_*V*_*^GABA^* in mice treated with saline7D or CFA7D. Two-way ANOVA with Bonferroni post-tests [Time × group interaction, *F*(1,21) = 1.403 and *P* = 0.2495]. **(L,M)** The alterations in action potential properties in **(L)**, rheobase; *t*_38_ = 1.558 and *P* = 0.127; **(M)** *V*_*rest*_ (mV); t_38_ = 0.526 and *P* = 0.6. **(N)** Sample traces of the mEPSCs from saline7D or CFA7D groups. **(O,P)** Summarized statistical data of **(O)** the frequency; *t*_45_ = 1.26 and *P* = 0.21; **(P)** the amplitude; *t*_45_ = 0.72 and *P* = 0.47 of CFA7D compared with saline7D mice. Action potential parameters were estimated from the initial evoked spike. *n* = 22–25 neurons per group. All data are presented as mean ± SEM. *P < 0.05, **P < 0.01, and ***P < 0.001; ns, not significant.

To visualize GABAergic neurons, glutamic acid decarboxylase *(GAD)-Cre* mice were crossed with *Ai14 (RCL-TDT)* mice to produce transgenic mice with red tdTomato-expressing GABAergic (GAD-tdTOM) neurons (Figure 2A) for visualized whole-cell patch-clamp recording in acute brain slices. The ZI_*V*_*^GABA^* firing rate elicited by injecting depolarizing currents was compared in saline1D versus CFA1D mice. Under the current-clamp recording mode, we observed a decline in the current-evoked action potentials of the ZI_*V*_*^GABA^* (Figure 2B) and significant differences in the firing between saline1D and CFA1D groups (Figure 2C). However, there were no differences in the rheobase (Figure 2D) and the resting membrane potential (*V*_*rest*_) (Figure 2E) between these two groups. Moreover, we have tested the excitatory synaptic transmission of ZI_*V*_*^GABA^* under voltage-clamp conditions. We found that the frequency of miniature excitatory postsynaptic currents (mEPSCs) was decreased (Figures 2F,G), but not the amplitude (Figure 2H) in CFA1D neurons compared with saline1D. Our results are consistent with previous observations that the ZI_*V*_*^GABA^* activity was reduced in the SCI (Whitt et al., 2013; Moon et al., 2016) and CCI (Moon and Park, 2017; Wang et al., 2020) pain models. However, these changes in ZI_*V*_*^GABA^* activity were not found in CFA7D mice compared to saline7D (Figures 2I–P).

### Manipulation of ZI_*V*_*^GABA^* Activity Regulates Pain Perception and Anxiety-Like Behaviors

**FIGURE 3:**
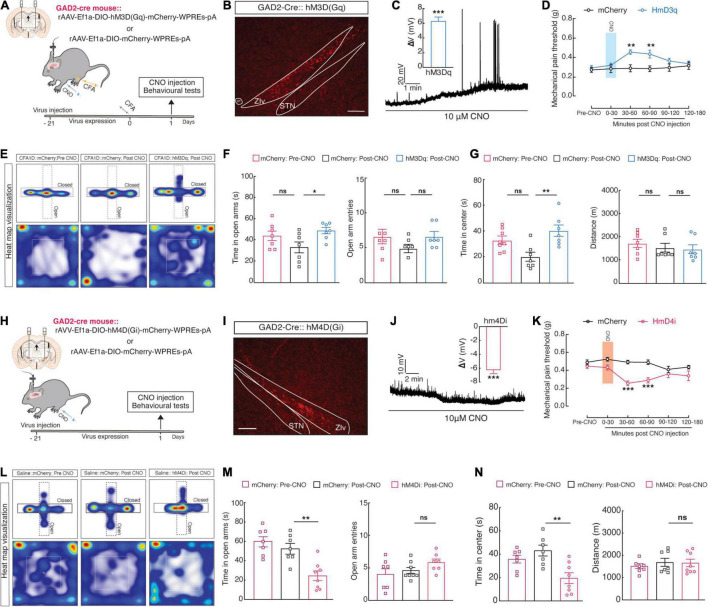
Manipulation of ZI*^GABA^* activity regulates pain perception and anxiety-like behaviors. **(A)** Schematic showing unilateral injection of ZI_*V*_ for chemogenetic manipulation and **(B)** diagram of viral injection of AAV-DIO-hM3Dq-mCherry (hM3Dq-mCherry) in the ZI_*V*_ of *GAD2-Cre* mice. The scale bar is 200 μm. **(C)** A representative trace displays that bath application of CNO (10 μM) depolarized ZIv*^GABA^*, and statistics (right) show the average magnitude of depolarization (One sample *t*-test, *t*_4_ = 11.45 and *P* = 0.03). **(D–G)** Chemogenetic activation of ZIv*^GABA^* in *GAD2-Cre* mice affects anxiety-like behaviors in acute pain (Time in open arms: mCherry *vs.* hM3Dq, *P* = 0.0469, time in the center; mCherry *vs.* hM3Dq, *P* = 0.0036). **(D)** Mechanical pain threshold; two-way ANOVA with Bonferroni post-tests [Time × group interaction, *F*(5,65) = 2.235 and *P* = 0.01612]. **(E)** Representative animal heat tracks in mCherry *vs.* hM3Dq mice in EPM and OFT. **(F)** Time in open arms; ordinary one-way ANOVA with Turkeys’ post-test (mCherry *vs.* hM3Dq, *P* = 0.0469). **(G)** Time in the center; ordinary one-way ANOVA with Turkeys’ post-test (mCherry *vs.* hM3Dq, *P* = 0.0015). *n* = 7 mice per group. **(H)** Schematic of viral injection in the ZI_*V*_ for chemogenetic manipulation and **(I)** diagram of bilateral injection of Cre recombinase-dependent AAV virus expressing hM4Di or mCherry AAV-DIO-hM4Di-mCherry (hM4Di-mCherry) into the ZI_*V*_ of *GAD2-Cre* mice. The scale bar is 200 μm. **(J)** A representative trace from a whole-cell current-clamp electrophysiological recording shows that bath application of CNO (10 μM) hyperpolarizes ZIv*^GABA^*. Statistics (right) show the average magnitude of hyperpolarization (One sample *t*-test, t_6_ = 11.71 and *P* < 0.0001). **(K–N)** Chemogenetic inhibition of ZIv*^GABA^* in *GAD2-Cre* mice induces anxiety-like behaviors and pain in naïve mice. **(K)** Mechanical pain threshold; two-way ANOVA with Bonferroni post-tests [Time × group interaction, *F*(5,65) = 3.199 and *P* = 0.0121]. **(L)** Representative animal heat tracks in mCherry *vs.* hM4Di mice in EPM and OFT. **(M)** Time in open arms; ordinary one-way ANOVA with Turkeys’ post-test (mCherry *vs.* hM4Di, *P* = 0.0036). **(N)** Time in the center; ordinary one-way ANOVA with Turkeys’ post-test (mCherry *vs.* hM4Di, *P* = 0.0024). *n* = 7–8 mice per group. All data are expressed as mean ± SEM. *P < 0.05, **P < 0.01, ***P < 0.001, and ns, not significant.

Consistent with previous studies in SCI (Whitt et al., 2013) and CCI (Wang et al., 2020) mouse models, we observed a reduction in ZI_*V*_*^GABA^* activity in CFA1D mice. Moreover, the GABAergic inhibitory pathway significance in preserving chronic pain has been shown before, as pain sensation was inverted by a GABA*^A^* receptor agonist (Moon and Park, 2017). Therefore, we examined whether the ZI_*V*_*^GABA^* activation could relieve anxiety-like behaviors in pain. Using *Cre*-dependent expression of chemogenetic excitatory hM3Dq (AAV-DIO-hM3Dq-mCherry) in the ZI_*V*_ and injection of its ligand, CNO (i.p.), we selectively activated ZI_*V*_*^GABA^* in *GAD2-Cre* mice (Figures 3A,B). To examine the function of the chemogenetic virus, the ZIv*^GABA^* in the brain slices that expressed the mCherry were recorded under the current-clamp mode; we found that resting membrane potential (*V*_*rest*_) of the ZI_*V*_*^GABA^* was depolarized activity had to action potentials after CNO (10 μM) administration (Figure 3C). Behavioral testing was performed 1-day post-CFA injection. Following CNO injection, we found a significant increase in mechanical pain thresholds (Figure 3D) and increased time in open arms in the EPM and time in the center of the OFT (Figures 3E–G) in the hM3Dq-mCherry group compared to the mCherry group.

Additionally, in naïve mice, intra-ZI_*V*_ injection of *Cre*-dependent expression of chemogenetic inhibitory hM4Di (AAV-DIO-hM4Di-mCherry) in *GAD2-Cre* mice selectively inhibited ZI_*V*_*^GABA^* (Figures 3H,I). Using the same method described above, we found that the resting membrane potential (*V*_*rest*_) of the ZI_*V*_*^GABA^* injected with the hM4Di virus was hyperpolarized after CNO (10 μM) perfusion (Figure 3J). We discovered that these mice showed higher pain sensitivity (Figure 3K) and anxiety-like behaviors (Figures 3L–N) post-CNO injection. These results suggested that deceased ZI_*V*_*^GABA^* activity may be required and adequate for anxiety and acute pain progress induced by CFA injection.

### Increased Microglial Cell Activity and Engulfment of ZI_*V*_*^GABA^* Spines in CFA1D Mice

**FIGURE 4:**
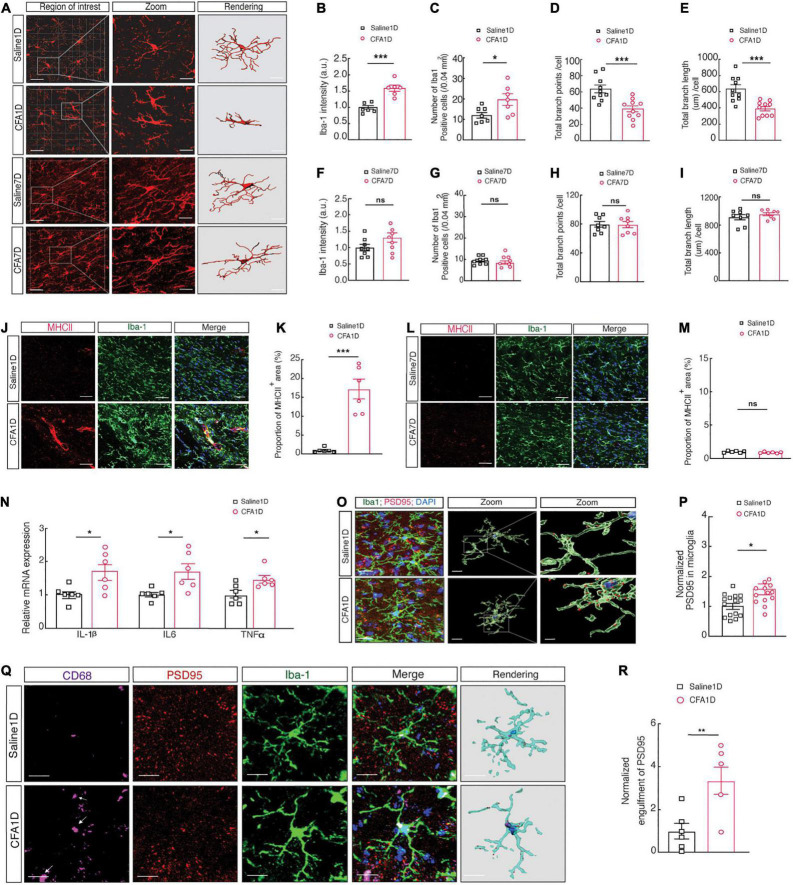
Increased microglial cell activity and engulfment of ZI_*V*_*^GABA^* spines in CFA1D mice. **(A)** Fluorescence images displaying Iba-1 immunostaining (red) and 3D reconstruction of microglia in the ZI_*V*_ of saline1D-, CFA1D-, saline7D- and CFA7D mice. The scale bars are 40 μm (overview) and 10 μm (inset and rendering). **(B,C)** Statistical data for the number of Iba-1^+^ cells per cubic millimeter (*t*_10_ = 2.46 and *P* = 0.029) and Iba-1 intensity (*t*_10_ = 5.42 and *P* = 0.003) in the ZI_*V*_ from saline1D- and CFA1D mice. **(D,E)** Summary data of microglia branches endpoints (*t*_18_ = 4.07 and *P* = 0.0007) and total process lengths per cell of Iba-1^+^ microglia (*t*_18_ = 4.43 and *P* = 0.0003) in ZIv from saline1D and CFA1D mice. **(F,G)** Statistical data for the number of Iba-1^+^ cells per cubic millimeter (*t*_14_ = 0.9354 and *P* = 0.365) and Iba-1 intensity (*t*_14_ = 1.765 and *P* = 0.0994) in the ZI_*V*_ from saline7D- and CFA7D mice. **(H,I)** Summary data of total microglia endpoints (*t*_14_ = 0.06 and *P* = 0.953), and total branches lengths per cell of Iba-1^+^ microglia (*t*_14_ = 0.965 and *P* = 0.3505) in ZIv from saline7D and CFA7D mice. **(J,K)** Representative images (left) and quantitative analyses (right) of immunostaining for Iba-1 (green) and MHCII (red) (*t*_10_ = 6.178 and *P* = 0.0001) in the ZI_*V*_ of saline1D- or CFA1D-treated mice. Scale bar, 10 μm. **(L,M)** Representative images (left) and quantitative analyses (right) of immunostaining for Iba-1 (green) and MHCII (red) (*t*_10_ = 1.799 and *P* = 0.1022) in the ZI_*V*_ of saline7D- or CFA7D-treated mice. Scale bar, 10 μm. **(N)** qPCR analysis of IL-1β (*t*_10_ = 2.719 and *P* = 0.0216), IL6 (*t*_10_ = 2.901 and *P* = 0.0158), and TNFα (*t*_10_ = 2.740 and *P* = 0.0208) mRNA in ZI_*V*_ tissues from saline1D and CFA1D mice. **(O)** Illustrative images and 3D reconstruction surface rendering of Iba-1^+^ microglia (green) including PSD95^+^ puncta (red) and DAPI (blue) in the ZI_*V*_ from saline1D- and CFA1D- treated mice. Scale bars, 10 μm (overview) and 2 μm (inset and rendering). **(P)** PSD95^+^ puncta’s quantification of microglia in slices (*t*_26_ = 2.71 and *P* = 0.011, *n* = 14–16 slices per group from four mice) as showed in **(O)**. **(Q,R)** Representative images (left) and quantitative analyses (right) of immunostaining for Iba-1 (green), CD68 (purple), and PSD95 (red) (*t*_10_ = 3.212 and *P* = 0.0093) in the ZI_*V*_ of saline1D- or CFA1D-treated mice. Scale bar, 10 μm. All data are presented as mean ± SEM. *n* = 6–10 slices per group from three mice. Unpaired-*t* test. *P < 0.05; **P < 0.01, and ***P < 0.001; ns, not significant.

Moreover, since microglia involvement in pain development is a well-established (Shan et al., 2007), we examined whether microglial morphology changes in the CFA1D and CFA7D conditions (Figure 4A). The morphological changes of microglia are strictly associated with their state of activation (Imai et al., 1996). Our results indicate that microglial morphology in CFA1D mice significantly altered the ZI’s, characterized by increased ionized calcium-binding adaptor molecule 1 (Iba-1) intensity (Figures 4B,C), which is a cytoplasmic protein limited to the microglia (Imai et al., 1996). Moreover, we observed a decrease in branch endpoints and microglial branch length per cell (Figures 4D,E). Interestingly, CFA7D mice did not significantly alter the ZI_*V*_’s microglial (ZIv*^Microglia^*) morphology, including microglial cell intensity, the process endpoints number, and the microglia processes length per cell (Figures 4F–I). Whether ZIv*^Microglia^* interacts with ZI_*V*_*^GABA^* and then contributes to the pathophysiology of inflammatory pain remains vague.

Microglia cells are critical in neuroinflammation, early development of pain, and promoting inflammatory processes by releasing different proinflammatory cytokines such as IL-1β, TNFα, and IL6 (Smith et al., 2012). Moreover, microglial reactivity is associated with MHCII induction in the acute inflammation condition (Hiremath et al., 2008). Subsequently, we found that the expression of proinflammatory cytokines, including IL-1β, TNFα, and IL6 (Figure 4N), accompanied by an increase in levels of MHCII (Figures 4J,K) in CFA1D mice is associated with the reactivity of microglial cells. The MHCII level did not alter in CFA7D mice compared to the saline7D (Figures 4L,M). The 3D reconstruction of microglia cell morphology showed abundant immunoreactive puncta of PSD95^+^, a marker for postsynaptic components, and Iba-1 labeled microglial processes colocalized in the ZI_*V*_ of CFA1D- and saline1D- mice (Figures 4O,P).

Moreover, the abundant immunoreactive puncta of PSD95 colocalized with Iba-1 labeled microglia in the ZI_*V*_ were increased in CFA1D compared to the saline1D mice (Figures 4Q,R). These results suggest a possible involvement of increased microglial engulfment of synapses in anxiety-like behavior in acute pain.

### Inactivation of ZI_*V*_*^Microglia^* Reverses Pain Sensitization and Anxiety-Like Behaviors in CFA1D Mice

**FIGURE 5:**
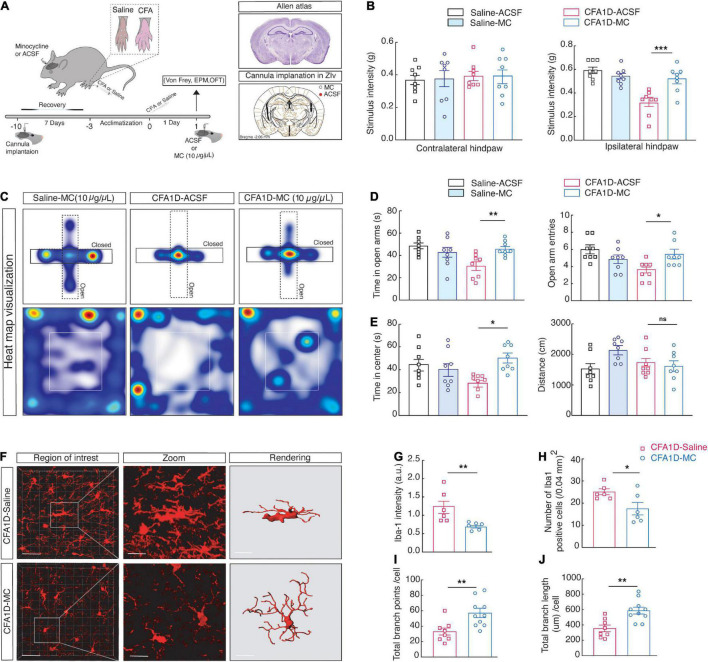
Inactivating microglial cells in ZI_*V*_ of CFA1D mice reverses anxiety-like behavior in pain and the dynamic modifications of microglial status. **(A)** Schematic diagram of the experiment and cannula implantation in ZI_*V*_. **(B)** Stimulus intensity of the ipsilateral (*P* = 0.002) and contralateral (*P* > 0.9) hind paws CFA1D mice during intra- ZI_*V*_ injection of minocycline (10 μg/μL) or ACSF, Ordinary one-way ANOVA with Bonferroni’s multiple comparisons tests. **(C–E)** Heat map visualization and statistics data from EPM [Time in open arm (*P* = 0.0024), open-arm entries] and OFT [Time in the center (*P* = 0.0114) and distance traveled] apparatuses in CFA1D mice during intra- ZI_*V*_ injection of minocycline (10 μg/μL) or ACSF, *n* = 8 mice per group. **(F)** Fluorescence images of Iba-1^+^ cells from CFA1D mice with saline or minocycline, scale bar, 20 μm. Sections show a detailed view of the white box; scale bar, 10 μm. **(G–J)** Statistical data for the Iba-1^+^ cell’s number, Iba-1 intensity, endpoints, and process lengths per cell. *n* = 6 slices per group from three mice. **(G,H)** Statistical data for the number of Iba-1^+^ cells per cubic millimeter (Iba-1^+^ cells; *t*_10_ = 2.448 and *P* = 0.0344) and Iba-1 intensity (Iba-1 intensity; *t*_10_ = 3.29 and *P* = 0.0081) in the ZI_*V*_ from saline- and minocycline treated CFA1D mice. **(I,J)** Summary data of microglia total branches endpoints (*t*_16_ = 3.054 and *P* = 0.0076) and all process lengths per cell of Iba-1^+^ microglia (*t*_16_ = 3.65 and *P* = 0.0022) in ZI_*V*_ from saline- and minocycline treated CFA1D. All data are expressed as mean ± SEM. *n* = 6–11 slices per group from three mice. *P < 0.05, **P < 0.01, ***P < 0.001; ns, not significant.

Given the increased ZI_*V*_*^Microglia^* activity in CFA1D animals, we consequently inactivated microglia to examine the anxiety-like behaviors in pain of CFA1D mice (Figure 5A). Intra- ZI_*V*_ injection of (10 μg/μL) minocycline in the contralateral hemisphere prevented ZI_*V*_^Microglia^ activation in CFA1D mice. Compared with the ACSF group, CFA mice with minocycline treatment showed a significant decrease in pain (Figure 5B) and anxiety-like behavior (Figures 5C–E). Minocycline inversed the decline of mechanical threshold in CFA1D mice in contralateral hind paw (Figure 5B) and escalated time in open arms (Figure 5D) of EPM and time in the center (Figure 5E) of OFT. Additionally, locomotor activity was not affected by minocycline treatment in this study.

Additionally, in morphological studies, minocycline microinjection significantly blocked the ZI’s microglial morphological changes after CFA injection (Figure 5F), characterized by a significant decrease in the Iba-1 intensity (Figures 5G,H). Moreover, the reduction of the number of endpoints and process length per cell in CFA1D mice was significantly retreated by minocycline in CFA1D mice (Figures 5I,J). These findings suggest that the reaction of ZI_*V*_*^Microglia^* in CFA1D mice mediates the hypersensitivity and anxiety-like behaviors caused by CFA-induced inflammation.

### Blockade of Microglial Reactivation Affects GABAergic Neuronal Activity and Microglial Engulfment of Spines in CFA1D Mice

**FIGURE 6:**
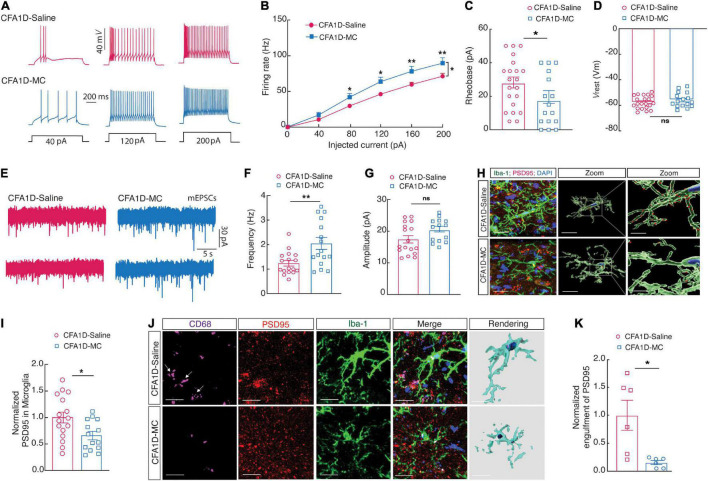
Blockade of microglial activation affects ZI_*V*_*^GABA^* activity and microglial engulfment of ZI_*V*_ neuronal spines in CFA1D mice. **(A)** Sample traces and **(B)** summarized statistical data for action potential firing recorded from ZI_*V*_*^GABA^* in CFA1D mice treated with saline or minocycline. Two-way ANOVA with Bonferroni post-tests [Time × group interaction, *F*(5,175) = 3.78 and *P* = 0.0028]. **(C,D)** The alterations in action potential properties in **(C)** rheobase (*t*_35_ = 0.98 and *P* = 0.33); **(D)** *V*_*rest*_ (mV) (*t*_26_ = 0.98 and *P* = 0.33); **(E)** sample traces of the mEPSCs from CFA1D mice treated with saline or minocycline. **(F,G)** Summarized statistical data of **(F)** the frequency (*t*_31_ = 3.05 and *P* = 0.0046); **(G)** the amplitude (*t*_30_ = 1.92 and *P* = 0.06) of CFA1D mice treated with saline or minocycline. Action potential parameters were estimated from the initial evoked spike. *n* = 16–21 neurons per group. **(H)** Representative images and quantification of Iba-1^+^ microglia (green) containing PSD95^+^ puncta (red) in the ZI_*V*_ from CFA-treated mice treated with minocycline or saline. Scale bars, 10 μm (overview) and 2 μm (inset and rendering). **(I)** PSD95^+^ puncta’s quantification of microglia in slices (*t*_26_ = 2.55 and *P* = 0.016). *n* = 14–16 slices per group from four mice. **(J,K)** Representative images (left) and quantitative analyses (right) of immunostaining for Iba-1 (green), CD68 (purple), and PSD95 (red) in the ZI_*V*_ of saline1D- or CFA1- mice treated with minocycline slices (*t*_10_ = 3.117 and *P* = 0.0109). Scale bar, 10 μm. Unpaired *t*-test. All data are presented as mean ± SEM. *P < 0.05 and **P < 0.01; ns, not significant.

The ZI contains GABAergic neurons and regulates various CNS functions, including the nociception (Petronilho et al., 2012). We investigated whether the blockade of reactive ZI_*V*_*^Microglia^* affects the decreased ZI_*V*_*^GABA^* activity in CFA1D mice, as shown in (Figure 6). We found that the ZI_*V*_*^GABA^* activity was reversed in the CFA1D mice after minocycline treatment (50 mg/kg, i.p.), which was manifested as reversed current-evoked action potentials (Figures 6A,B), and the rheobase for the action potentials (Figure 6C). However, there was no significant change in the *V*_*rest*_ (Figure 6D). In addition, we also examined the mEPSCs in the CFA1D mice after both treatments (Figure 6E), and we found the frequency (Figure 6F) but not the amplitude (Figure 6G), was recovered in the minocycline treatment compared with the saline1D treatment group.

Moreover, 3D reconstruction displayed that abundant immunoreactive puncta of PSD95^+^ and Iba-1 labeled microglial processes localized in the ZI_*V*_ of CFA1D mice were inverted after microinjection of minocycline (Figures 6H,I). Together, these results indicated that the enhanced ZI_*V*_*^GABA^* might result from microglial engulfment in CFA1D mice, and the suppression of the microglial activation could reverse the ZI_*V*_*^GABA^*.

To further examine the interactions of the microglial processes and dendritic spines of ZI_*V*_*^GABA^*, we performed cell-type-specific sparse labeling by injection of AAV-sparse-CSSP-YFP-8E3 virus into ZI_*V*_ in *GAD2-Cre* mice to specifically label neuronal dendritic spines of ZI_*V*_*^GABA^*. The increased level of Iba-1 is significantly correlated with the levels of CD68 (Cao et al., 2021). Using confocal imaging and 3D surface rendering, we showed that abundant immunoreactive puncta of PSD95, CD68, and Iba-1 labeled microglial processes colocalized in the ZI_*V*_ of CFA1D mice but not saline-treated mice (Figures 4Q,R), and these phenotypes were reversed upon minocycline treatment (Figures 6J,K).

**FIGURE 7:**
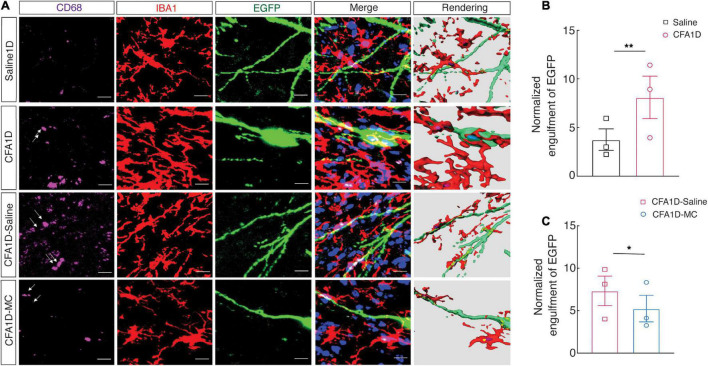
The interactions of the microglial processes and dendritic spines of ZI_*V*_*^GABA^*. **(A)** Representative immunofluorescence images and 3D rendering of CD68 (purple), Iba-1 (red), and EGFP (green) of AAV-sparse-CSSP-YFP-8E3 virus into ZI_*V*_ in the indicated groups. **(B)** Quantification of EGFP^+^ dendritic spines (green) containing Iba-1^+^ microglia (red) in the ZI_*V*_ from CFA1D and saline mice (*t*_10_ = 3.21 and *P* = 0.0093). **(C)** Quantification of EGFP^+^ dendritic spines (green) Iba-1^+^ microglia (red) in the ZI_*V*_ from saline and minocycline treatment of CFA1D mice (*t*_10_ = 3.117 and *P* = 0.0109). Scale bars, 10 μm. Unpaired *t*-test. *P < 0.05 and **P < 0.01; ns, not significant.

Moreover, we performed cell-type-specific sparse labeling by injection of AAV-sparse-CSSP-YFP-8E3 virus into ZI_*V*_ in *GAD2-Cre* mice to specifically label neuronal dendritic spines of GABAergic neurons (Figure 7A). We found an increase in the engulfment of EGFP within microglial lysosomes in the CFA1D mice compared to controls (Figure 7B). However, the engulfment of EGFP was recovered after treatment with minocycline (Figure 7C). Our data further suggest that reactive microglia mediate synapse engulfment and anxiety-like behavior in pain.

## Discussion

A growing body of evidence shows that immune system dysfunctions could play the primary role in developing a pain sensitivity (Calvo et al., 2012). Significant aspects of the inflammatory signaling during mood disorders are mediated by microglial cells (Pfau et al., 2018), which promptly activate in neurological diseases and become extremely motile, secreting inflammatory cytokines, migrating to the lesion area, and phagocytizing cell debris or damaged neurons (Fu et al., 2014). Despite the central role of microglial cells in the neuronal activity regulation (Inoue, 2006), whether and how microglia contribute to anxiety-like behaviors in pain is still vague. The present study is the first to report the microglial engulfment of dendritic spines in ZI_*V*_ in anxiety-like behaviors in a mouse model of acute pain.

As the former study showed, mice with CFA inflammatory pain revealed meaningful anxiety-like behavior 4 h following injection, which vanished on the day seven (Zhou W. et al., 2021) and again present after 3 weeks (Jin et al., 2020). We demonstrated that CFA injection is reliable for inducing inflammatory pain in a time course of 1, 3-, 7-, and 10-days post-injection. Also, CFA1D mice displayed significant pain and anxiety-like behaviors. Withal significant pain hypersensitivity in CFA7D mice, we did not find anxiety-like behavior. Hence, the anxiety-like behavior in the acute pain mouse model was established through CFA injection.

Previous studies also reveal that neuropathic pain is associated with a reduction in neuronal activity of the ZI*^GABA^* in a rat nerve CCI (Moon and Park, 2017) and a SCI model (Moon et al., 2016). The infusion of a GABAergic drug into the ZI_*V*_ could restore its inhibitory action and recover neuropathic pain (Moon et al., 2016). Also, GABA*^A^* receptor agonists reduced pain hypersensitivity and increased the firing rate of ZI*^GABA^* in the CCI mice (Moon and Park, 2017). Hence, we hypothesized ZI as one of the main areas in the pathology of inflammatory pain and concentrated on the role of the ZI neural alterations during anxiety-like behaviors in pain. Reduced GABAergic phasic inhibitory transmission is believed responsible for initiating and maintaining the neuropathic pain (Iura et al., 2016). Our results demonstrated that ZIv*^GABA^* activity decreased after CFA1D injection, accompanied by the expression of anxiety-like behaviors in pain. This hypothesis that decreased ZI_*V*_*^GABA^* causes anxiety-like behaviors in pain might be further assessed experimentally using different pain stimuli. Our results show that pain-related anxiety-like behaviors were prevented upon treatment with minocycline. So, microglial reactivation might contribute to inflammation-induced hypoactivity of ZI_*V*_*^GABA^*. These initial results support our subsequent studies using a chemogenetic approach to functionally manipulate the activity of the ZI_*V*_*^GABA^* to address its role in anxiety-like behaviors in pain.

Microglial reactivation leads to abnormal neurons in the amygdala (Munshi et al., 2020) and the dorsal striatum (Han et al., 2020), triggering anxiety-like behavior. Previous studies have demonstrated that ZI’s neuronal plastic changes are associated with astrocyte-induced synapse modulation. MCS treatment modulated the ZI’s astroglia and synaptic changes in the primary motor cortex (M1) and reduced neuropathic pain in nerve-injured rats (Myeounghoon et al., 2020). An MCS-induced neuropathic pain model has investigated the relationship between pain alleviation and ZI neuroplasticity. MCS modulates the astrocyte activities in the ZI and synaptic changes in the M1 (Cha et al., 2020). Prominently, chemogenetic activation of contralateral ZI_*V*_*^GABA^* in CFA1D mice reverses anxiety-like behaviors in pain. However, chemogenetic inhibition of ZI_*V*_ inhibitory neurons induced the development of anxiety-like behaviors in pain. Our experiments align with previous studies showing that the ZI-Po circuit and cannabinoids regulate CCI-induced neuropathic pain hypersensitivity by decreasing ZI’s neuronal activity (Wang et al., 2020).

Microglial morphotypes are different in healthy and pathological situations; surveilling, satellite, juxtavascular, and amoeboid microglia in a healthy brain (Augusto-Oliveira et al., 2022). Following treatment with microglial manipulators, microglial cells modify neuronal and synaptic activities to alter pain behavior (Wang et al., 2019; Xin, 2019). Besides the antibiotic properties of minocycline, it enhances retinoic acid signaling, which is involved in pleiotropic neuroprotective and anti-inflammatory properties in the CNS. (Regen et al., 2016; Clemens et al., 2018). Minocycline can cross the blood-brain barrier (BBB) and prevent microglial-related inflammation (Garrido-Mesa et al., 2013). Several studies have been made to clarify how minocycline ameliorates different comorbidity of pain (Rojewska et al., 2014; Sun et al., 2016) and anxiety (Rooney et al., 2020)—the direct connection between microglial activation and pain development after nerve injuries. Also, in a previous study, minocycline reduced microglial activation, nociceptive behavior, and glutamatergic neuronal activity in the primary somatosensory cortex (S1BF) (Wang et al., 2019).

Despite the role of activated microglial cells in neuroinflammation and pain regulation, the neurobiological mechanisms underlying anxiety-like behavior in pain remain unclear. By staining with Iba-1 of ZI_*V*_*^Microglia^*, we discovered noticeable microglial activation after CFA1D injection, whereas minocycline lessened the activated state of microglial cells in CFA1D mice.

Our results indicate that treatment with minocycline before behavioral experiments moderated pain and anxiety-like behaviors in pain in CFA1D mice. Moreover, following CFA1D injection, ZI_*V*_*^Microglia^* were significantly activated, and minocycline reduced anxiety-like behaviors in pain. and reversed decreased ZI_*V*_*^GABA^* activity. These findings propose a satisfactory and essential role of decreased ZI_*V*_*^GABA^* activity in improving anxiety-like behaviors in pain. Consistent with our results, preceding studies discovered that minocycline decreased the progress hypersensitivity after peripheral nerve injury (Raghavendra et al., 2003; Ledeboer et al., 2005). Also, microglial morphological changes such as density and a complicated process were observed in the medial prefrontal cortex (Caetano et al., 2017), hippocampus (Duarte et al., 2019), and amygdala (Wohleb et al., 2011) of animal models of anxiety.

It has also been proven that the complement pathway plays a role in the microglial engulfment of dendritic spines in response to disease and early life development (Dykman et al., 1997; Vertes, 2006; Hong et al., 2016; Qing et al., 2018). During development, microglia interact with synapses to change their functions and structures in the brain. For example, microglial development engulfs synapses and prompts synaptic pruning in critical developmental points (Stevens et al., 2007). Moreover, early-life inflammation reduced the glutamatergic neural activity in the anterior cingulate cortex (ACC) and augmented the dendritic spines’ elimination and formation (Cao et al., 2021). A rapid increase follows microglial phagocytosis in producing reactive oxygen species (ROS), which facilitate phagocytic contents degradation and may have a detrimental impact on surrounding neurons (Andrews et al., 2008). Also, a chronic unpredictable stress model (CUS) model shows that stress exposure generates microglial cell engulfment of synapses which causes anxiety and depression-like behavior (Bian et al., 2012). However, the mechanism behind microglia-mediated synapse remodeling is still not well understood.

Here, we demonstrate how inflammation causes alterations in states of microglial cells in a manner that promotes anxiety-like behaviors in pain by increasing the extent of microglial engulfment of spines in the ZI_*V*_. These results suggest that acute inflammatory pain leads to increased microglial engulfment of synapses following acute inflammation in CFA1D mice. Interestingly, this microglial engulfment of synapses in CFA1D mice was reversed by minocycline treatment.

The peripheral neuroinflammation characterized by activation of microglial cells and astrocytes could generate the central sensitization (Ji et al., 2014). The activation of glial cells releases different proinflammatory cytokines (e.g., IL-1β, TNFα), affecting the inhibitory and excitatory synapses (Latremoliere and Woolf, 2009). Moreover, microglial reactivity is associated with MHCII induction in the acute inflammation condition (Hiremath et al., 2008). Our results showed that CFA1D treatment induces microglial activation in the ZI_*V*_ by upregulation of proinflammatory cytokines, including IL-1β, TNFα, and IL6 from microglia, accompanied by increased levels of MHCII in the ZI_*V*_ of CFA1D mice is associated with the reactivity of microglial cells. However, the indicators of microglial activation recovered in CFA7D mice.

Complement cascade’s dysregulation drives inflammatory reactions, associated with the pathogenesis of numerous neurodegenerative disorders (Hong et al., 2016; Clarke et al., 2019). This cascade mediates immune system activation to reduce phagocytosis. Specifically, microglial cells regulate synapse density by modulation of extracellular matrix and phagocytic elimination of synapse (Nguyen et al., 2020). Microglial cells are involved in various brain pathogenesis via their roles as central regulators of the neuroinflammation (Prinz et al., 2011). Phagocytic cells such as microglia and macrophages perform phagocytosis (Fu et al., 2014). Microglial engulfment of synapses contributes to cognitive dysfunction, such as depressive behaviors (Kang et al., 2012; Liu et al., 2017). Moreover, microglial-induced apoptosis is possibly responsible for hyperalgesia alterations during the CFA-induced inflammation (Baniasadi et al., 2020).

Finally, we showed that abundant immunoreactive puncta of PSD95, CD68, and Iba-1 labeled microglial processes colocalized in the ZI_*V*_ of CFA1D mice, and these phenotypes were reversed upon minocycline treatment.

Moreover, we found an increase in the engulfment of EGFP within microglial lysosomes in the CFA1D mice, which recovered after treatment with minocycline. Our data further suggest that reactive microglia mediate synapse engulfment and anxiety-like behavior in pain.

In summary, our study showed that CFA1D induced alterations in the state of microglial cells in the ZI_*V*_, leading to decreased ZI_*V*_*^GABA^* excitability. This microglia activation promoted excessive engulfment of spines that decreased ZI_*V*_*^GABA^* activity, resulting in inadequate activation of ZI_*V*_*^GABA^* neurons, promoting anxiety-like behaviors in pain. Studying the interaction between neurons and microglia requires extensive and systemic research. Our study has specified evidence that maladaptation of GABAergic neurons contributes to anxiety-like behaviors in pain and might be mediated by ZIv’s microglial cells. As pharmacological options for treating anxiety-like behavior in pain remain relatively inadequate, these findings raise the possibility of developing pharmacological targeting of microglial cells for its treatment.

Together, the microglial activation promoted excessive engulfment of spines that decreased ZI_*V*_*^GABA^* activity, resulting in insufficient inhabitation of ZI_*V*_*^GABA^* and promoting the development of anxiety-like behavior in pain.

## Data Availability Statement

The raw data supporting the conclusions of this article will be made available by the authors, without undue reservation.

## Ethics Statement

The animal study was reviewed and approved by Animal Care and Use Committee of the University of Science and Technology of China.

## Author Contributions

ZF, ZZ, and YJ designed research. ZF, AL, and PC performed research, analyzed data, and wrote the manuscript. YM and YJ funding acquisition. ZZ and YJ supervised the study. All authors listed have made a substantial, direct, and intellectual contribution to the work, and approved it for publication.

## Conflict of Interest

The authors declare that the research was conducted in the absence of any commercial or financial relationships that could be construed as a potential conflict of interest.

## Publisher’s Note

All claims expressed in this article are solely those of the authors and do not necessarily represent those of their affiliated organizations, or those of the publisher, the editors and the reviewers. Any product that may be evaluated in this article, or claim that may be made by its manufacturer, is not guaranteed or endorsed by the publisher.
